# Supplementary material for: Methods for Drainage of Distal Malignant Biliary Obstruction after ERCP Failure: A Systematic Review and Network Meta-Analysis
Source: Cancers (Basel). 2022 Jul 5;14(13):3291. doi: 10.3390/cancers14133291 (PMC9266204; doi:10.3390/cancers14133291)
Supplement: Supplementary file 1 [file cancers-14-03291-s001.zip › cancers-1760158-supplementary.pdf]

cancers

Methods for Drainage of Distal Malignant Biliary Obstruction after ERCP Failure: A Systematic Review and Network Meta-Analysis

Antonio Facciorusso [1,2], Benedetto Mangiavillano [3], Danilo Paduano [3], Cecilia Binda [4,*], Stefano Francesco Crinò [2], Paraskevas Gkolfakis [5], Daryl Ramai [6], Alessandro Fugazza [7], Ilaria Tarantino [8], Andrea Lisotti [9], Pietro Fusaroli [9], Carlo Fabbri [4] and Andrea Anderloni [7]

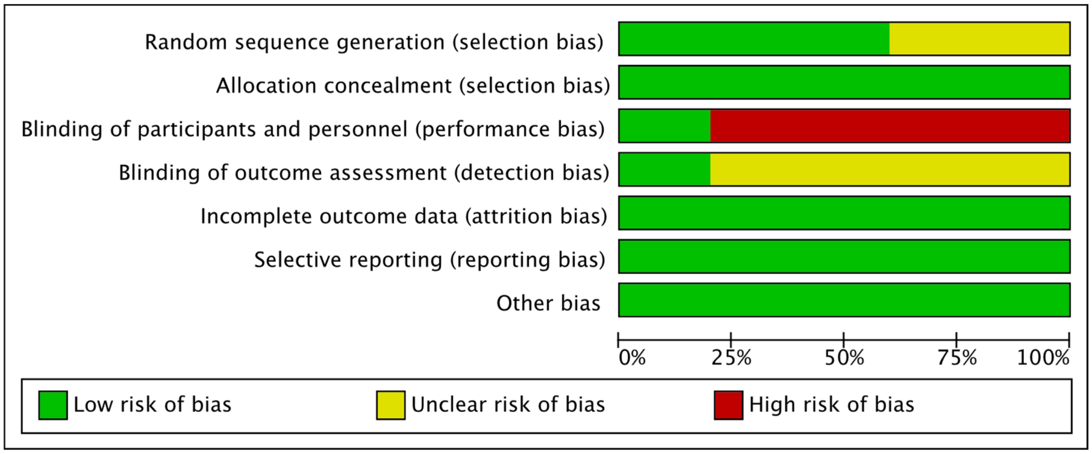

Figure S1. Risk of bias graph of the included trials.

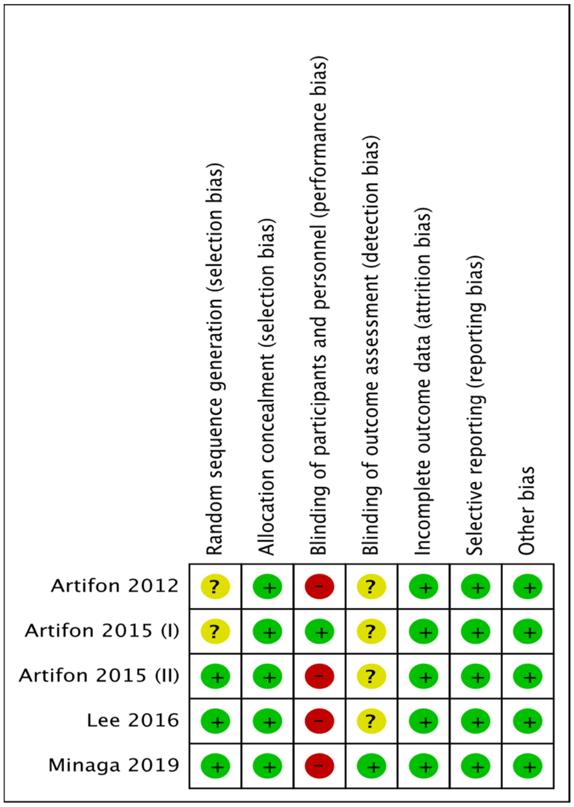

Figure S2. Risk of bias summary of the included trials.

Table S1. Results of network meta-analysis concerning technical success rate and moderate/severe adverse event rate.

	**Technical success rate**		**Moderate-Severe adverse event rate**	
	Risk Ratio (95% CI)	Quality of Evidence	Risk Ratio (95% CI)	Quality of Evidence
		**All treatments vs. PTBD**		
EUS-CD	1.02 (0.96-1.08)	Low	0.45 (0.21-2.03)	Low
EUS-HG	1.03 (0.97-1.09)	Low	0.35 (0.18-1.47)	Low
Surgery	1.09 (0.87-1.38)	Low	0.50 (0.10-3.52)	Low
		**vs. EUS-CD**		
EUS-HG	1.01 (0.93-1.09)	Low	0.54 (0.26-1.88)	Low
Surgery	1.07 (0.85-1.34)	Low	1.26 (0.22-3.91)	Low
		**vs. EUS-HG**		
Surgery	1.06 (0.83-1.34)	Low	1.75 (0.16-5.03)	Low

Abbreviations: EUS-CD, Endoscopic ultrasound choledochoduodenostomy; EUS-HG, Endoscopic ultrasound hepatico-gastrostomy; PTBD, Percutaneous trans-hepatic biliary drainage.

Table S2. SUCRA ranking of treatments for technical success rate and moderate/severe adverse event rate.

**Technical success rate**		**Moderate-severe adverse event rate**	
Surgery	0.54	EUS-HG	0.62
EUS-HG	0.47	EUS-CD	0.51
EUS-CD	0.36	Surgery	0.48
PTBD	0.31	PTBD	0.18

Abbreviations: EUS-CD, Endoscopic ultrasound choledochoduodenostomy; EUS-HG, Endoscopic ultrasound hepatico-gastrostomy; PTBD, Percutaneous trans-hepatic biliary drainage.

Table S3. Pooled incidence of different adverse events in the included trials.

**Adverse Event**	**EUS-CD**						**PTBD**			**Surgical hepato-jejunostomy**	**EUS-HG**			
	**Artifon 2012**	**Artifon 2015**	**Lee 2016 (I)**	**Minaga 2019**	**Artifon 2015 (II)**	**Overall**	**Artifon 2012**	**Lee 2016**	**Overall**	**Artifon 2015**	**Lee 2016 (II)**	**Minaga 2019**	**Artifon 2015 (II)**	**Overall**
Bleeding	0 (0%)	1 (6.2%)	0 (0%)	0 (0%)	1 (4.1%)	3.4% (0%-7.3%)	1 (8.3%)	3 (9.3%)	9.1% (0.6%-17.6%)	1 (6.2%)	0 (0%)	0 (0%)	3 (12%)	2.9% (0%-6.9%)
Perforation	0 (0%)	0 (0%)	0 (0%)	0 (0%)	1 (4.1%)	3.1% (0%-6.7%)	0 (0%)	0 (0%)	0%	0 (0%)	0 (0%)	0 (0%)	0 (0%)	0%
Stent migration	0 (0%)	1 (6.2%)	0 (0%)	0 (0%)	0 (0%)	2.8% (0%-6.2%)	0 (0%)	0 (0%)	0%	0 (0%)	0 (0%)	1 (4.1%)	0 (0%)	2.4% (0%-5.8%)
Acute pancreatitis	0 (0%)	0 (0%)	1 (12.5%)	1 (4.3%)	0 (0%)	3.1% (0%-6.8%)	0 (0%)	0 (0%)	0%	0 (0%)	1 (4%)	0 (0%)	0 (0%)	2.4% (0%-5.8%)
